# Interaction between rhizobacterial community and host root metabolism influences poplar salt tolerance

**DOI:** 10.1128/msystems.00635-26

**Published:** 2026-06-17

**Authors:** Yangwenke Liao, Qian Yu, Tingting Chen, Rui You, Qingyue Zhang, Xiaogang Li

**Affiliations:** 1 State Key Laboratory of Tree Genetics and Breeding, Nanjing Forestry University 74584, Nanjing, China; 2 Co-Innovation Center for Sustainable Forestry in Southern China, Nanjing Forestry University 74584, Nanjing, China; Oak Ridge National Laboratory, Oak Ridge, Tennessee, USA

**Keywords:** plant-microbe interaction, rhizosphere bacterial community, root metabolism, poplar, salt stress

## Abstract

Soil salinization worldwide affects agroforestry, restraining output and functions of farmland and forest ecosystems. Soil microbiota play vital roles in plant growth and resistance to stress, but how plants interact with root microbiomes to fight adverse environments remains elusive. Here, we employed high-throughput sequencing to investigate the rhizobacterial community composition of three poplar varieties that were *Populus davidiana* × *P. bolleana Loucne* (SXY), *P. deltoides* × *P. euramericana* "Nanlin 895” (NL895), and *P. alba* × *P. glandulosa* “84K” (84K) under salt stress. Our results showed that no differences in growth parameters and damage indices were observed across varieties before treatments. Furthermore, SXY exhibited the highest salt tolerance, characterized by the highest growth parameters and lowest damage indices under salt stress, while NL895 was the most sensitive genotype. The 16S rRNA gene sequencing unveiled the lowest diversity and distinct composition in the rhizobacterial community of SXY compared to other varieties. SXY accumulated a higher abundance of *Pseudomonas*, *Pseudoxanthomonas,* and Rhizobiaceae in the rhizosphere, which showed positive correlations with host salt tolerance. Moreover, metabolomic analysis revealed higher levels of certain secondary metabolites in SXY roots than in the roots of the other varieties. Four metabolites, including D-threitol, maslinic acid, 4′,5-dihydroxy-7-methoxyflavanone, and trans-3-coumaric acid, were identified as key regulators that potentially mediate the enrichment of salt tolerance-associated rhizobacterial taxa. Our findings indicate an interaction between root metabolism and rhizosphere microbiome in poplar adaptation to salt stress, providing a theoretical basis for directional modulation of plant resistance under global change.

**IMPORTANCE:**

Agroforestry frequently encounters soil salinization that limits crop yields and ecosystem services. Soil microbiota plays an important role in plant adaptation to stress, but their interaction mechanisms with host roots remain unclear. Through combining high-throughput sequencing and root metabolome analysis, we unraveled the interactions between rhizobacterial communities and host root metabolism, as well as their role in plant adaptation to salt stress, providing new strategies for microbial application under global change.

## INTRODUCTION

Salinization derived from human farming practices impacts over one billion hectares of soils (1), which causes ion toxicity, osmotic stress, oxidative damage, organ senescence, and plant death and results in crop yield loss in agroforestry (2, 3). Strategies including planting salt-resistant species/varieties and applying soil amendments have been used to improve the ecological conditions of saline soils (4, 5). However, these approaches suffer from limitations such as long implementation cycles and potential environmental risks. Developing efficient and eco-friendly strategies to enhance plant stress tolerance is therefore of great significance for improving crop productivity on saline lands.

Numerous microbes living in soil have been demonstrated to play important roles in plant growth and stress resistance (6). Beneficial microbes like plant growth-promoting rhizobacteria (PGPRs), endophytic bacteria, and mycorrhizal fungi can help hosts adapt to environmental stress by enhancing water and nutrient uptake, synthesizing phytohormones, and forming biofilms (7). Soil microbiota exhibits multi-dimensional responses to environmental stress, such as shifts in soil biodiversity, changes in community assembly, and enrichment of specific groups (8–10). Thus, previous studies have identified some salt-responsive microbes and constructed synthetic communities (SynComs) based on them to modulate plant stress tolerance (11, 12). However, most of these cases focus on microbes but ignore the influence of plants, leading to unstable and/or short-lived effects of these microbial agents during application. A full understanding of the mechanisms of plant-microbe interactions under salt stress would accelerate the development of beneficial microbial applications.

Plants have evolved a set of survival strategies during long-term adaptation toward adverse environments, including interactions with soil microbiota by modulating root metabolism (13). Interplays between root metabolism and microbial communities under stress, which are highly dependent on plant genotype, have been reported in several species (14, 15). For instance, certain maize cultivars increase flavones in roots, which recruits rhizosphere Oxalobacteraceae to enhance nutrient uptake for survival under nitrogen deprivation (16). The *Arabidopsis* mutant *fer-*8, which lacks FERONIA receptor kinase, enriches rhizosphere *Pseudomonas fluorescens* and alters host resistance to pathogens by modulating root-derived reactive oxygen species (17). Silencing *MdGH3-2/12* in apple decreases root strigolactone levels, which inhibits drought resistance via regulating arbuscular mycorrhizal colonization (18). However, the mechanism by which plants shape soil microbiota via specific metabolic pathways under salt stress remains unclear.

Here, we used poplar (*Populus* spp.), a forestry species with salt tolerance that is capable of interacting with various groups of microbes, as our model system. We proposed hypotheses that (i) genotype-dependent root metabolism of each poplar variety determines a distinct rhizosphere microbial community under salt stress and (ii) the salt-tolerant poplar plants accumulate specific metabolites to enrich beneficial microbial taxa in the rhizosphere during adaptation to salt stress. To test these hypotheses, we employed three poplar varieties, *Populus davidiana* × *P. bolleana* Loucne (SXY), *P. deltoides* × *P. euramericana* “Nanlin 895” (NL895), and *P. alba* × *P. glandulosa* “84K” (84K), to explore the salt-tolerant mechanisms in plants. SXY and 84K come from section (sect.) *Leuce* of *Populus*, which are mainly distributed in North China, while NL895 belongs to sect. *Aigeiros*, which grows in South China. We investigated the salt tolerance of three poplar varieties, performed 16S rRNA gene sequencing and root metabolome analysis, and further analyzed the associations between plant salt tolerance, microbial community composition, and root metabolome profiles. We aimed to unravel how salt-tolerant plants interact with the rhizosphere microbial community by regulating root metabolism under salt stress.

## MATERIALS AND METHODS

### Plant and treatments

Stem cuttings of SXY, NL895, and 84K were subcultured *in vitro* in Murashige and Skoog (MS) medium supplemented with the same formula as described in our previous study (19), including 6-benzylaminopurine (6-BA, 4.0 mg L^−1^), naphthalene acetic acid (NAA, 0.4 mg L^−1^), thidiazuron (TDZ, 0.04 mg L^−1^), sucrose (25 g L^−1^), and agar (5.5 g L^−1^). After sprouting, the tissues were transferred to 1/2 MS medium with 6-BA (4.0 mg L^−1^), NAA (0.2 mg L^−1^), sucrose (25 g L^−1^), and agar (5.5 g L^−1^) and cultivated into plantlets under a 16/8 h light/dark period, with illumination intensity of 360 μmol m^−2^ s^−1^ and day/night temperatures of 23/23°C. Before acclimation, at least four plantlets of each variety were reserved for biomass measurement and assays of physiological indices to obtain the baseline values of these parameters. The rest of the poplar plantlets were acclimated for 12 h, transferred into sterilized nutrient substrate (Xiangzheng, Hunan, China) for 15 days of cultivation, and watered once weekly with sterile ddH_2_O, under conditions as follows: 16/8 h light/dark period, 25/20°C day/night temperatures, and 70% relative humidity.

The soil culture experiments were conducted as described in Fig. S1. Coastal saline soil was obtained from the Dongtai forest farm of Jiangsu Province (32°51'08"N, 120°19'09"E) near the sea with soil properties and salinity shown in Table S1, wherein 0.27% (wt/vol) NaCl concentration (low salinity) was non-lethal for poplar, but 0.42% (high salinity) was a lethal dose. Subsequently, 12 plantlets of each variety were transplanted into plastic pots sterilized with 75% ethanol. Each pot was filled with 1.5 kg of a saline soil-perlite mixture (4:1, vol/vol) and contained only one plantlet. All plants were placed in an individual plastic tray and cultured under the same conditions as those used in the acclimation stage. Plants were carefully watered with sterile ddH_2_O every 3 days to prevent water leakage from the bottom of the pots.

### Sample collection and physiological assays

Before transplanting (0 day post-transplanting [dpt]), four plantlets of each poplar variety were selected to measure the shoot and root biomasses and calculate the corresponding root/shoot (R/S) ratio based on the fresh weight. Meanwhile, leaf and root tissues were harvested for physiological assays. At 120 dpt, the above parameters were measured in poplar plantlets exposed to low salinity conditions, and root metabolome profiling was subsequently performed. Each biological replicate was generated by pooling tissues from three to four plantlets, and four independent biological replicates were set up for each variety under low salinity. Under high salinity, the survival rate of poplar plantlets was calculated at 50 and 120 dpt.

Total chlorophyll content of the poplar leaves pooled from at least three plantlets in each treatment was extracted by soaking fresh leaf samples in 95% ethanol in the dark for 72 h. Subsequently, the absorbance of the extracts was determined at 646 nm and 663 nm using a spectrophotometer (Beckman, Pasadena, CA, USA), which was subsequently used to calculate the chlorophyll content as described in a previous study (20). Three biological replicates were analyzed for each treatment.

Malondialdehyde (MDA) content, an indicator of lipid peroxidation, was evaluated by measuring thiobarbituric acid-reactive substances (TBARS), as described in our previous work (19). Leaf and root tissues (0.2 g) were homogenized with 1.8 mL of 0.1% (wt/vol) trichloroacetic acid (TCA) on ice, followed by centrifugation at 12,000 × *g* for 20 min. The supernatant was used for MDA quantification. For the thiobarbituric acid (TBA) assay, 1 mL of the supernatant was mixed with 1 mL of 20% (wt/vol) TCA containing 0.5% (wt/vol) TBA. The mixture was boiled for approximately 30 min and then immediately placed on ice to abort the reaction. Subsequently, the mixture was centrifuged at 12,000 × *g* for 10 min. The absorbance of the supernatant was measured at 532 nm, and the value for non-specific absorption was determined at 600 nm. The value for the MDA-TBA complex was calculated using an extinction coefficient of 155 mM^−1^ cm^−1^.

### Soil sampling, DNA extraction, and 16S rRNA gene sequencing

Plantlets of each poplar variety were randomly assigned to three groups. Bulk and rhizosphere soils were sampled from poplar plantlets at 120 dpt under low salinity, following our previous work (19). Soil samples without plant cultivation were collected as bulk soil samples. Rhizosphere soils were pooled from three to four plantlets for each group to form one biological replicate and thoroughly mixed to yield a composite sample. One plantlet from each pot was uprooted, retaining the soil attached to the root system. Excess soil was manually removed by gentle shaking, leaving a 2-mm-thick soil layer still attached to the roots. Roots were then transferred into sterilized 250-mL conical flasks containing 100 mL of sterile PBS and shaken at 220 rpm for 20 min at 4°C to prepare rhizosphere suspensions. Roots were then carefully sorted out from the flasks without damage, and the suspensions were centrifuged at 6,000 × *g* for 10 min at 4°C. After discarding the supernatant, rhizosphere microbial pellets were stored at −20°C for DNA extraction.

Total DNA was extracted from 0.3 g of bulk and rhizosphere soil samples (12 samples in total) using the Fast DNA spin kit for soil (MP Biomedicals, Santa Ana, CA, USA). The final concentration and quality of DNA were verified using a NanoDrop 5000 spectrophotometer (Thermo Scientific, Wilmington, DE, USA). The V3-V4 region of the 16S rRNA gene was amplified using the universal primer pair 338F (5′- ACTCCTACGGGAGGCAGCA-3′)/806R (5′-GGACTACNNGGGTATCTAAT-3′) (21). Sample-specific 7-bp barcodes were incorporated into the primers for multiplex sequencing. All PCR reactions were performed in 25 μL volumes containing 5 μL of 5× reaction buffer (Takara Bio, Otsu, Japan), 5 μL of 5× GC buffer, 2 μL of each dNTP (2.5 mM), 1 μL of each primer (10 μM), 2 μL of DNA template, 0.25 μL of Q5 DNA polymerase, and 8.75 μL of ddH_2_O. After purification with Vazyme VAHTS DNA clean beads (Vazyme, Nanjing, China), the pooled 16S rRNA gene amplicons were sequenced on the Illumina Novaseq 6000-PE250 191 platform (Thermo Scientific, Wilmington, USA) at Shanghai Personal Biotechnology Co., Ltd. (Shanghai, China).

Raw data from sequencing were analyzed using the QIIME2 pipeline (https://docs.qiime2.org/2019.7/tutorials/overview/) (22). Low-quality sequences (<200 bp in length or those not matching the primer and barcode) were removed, and the remaining sequences were aligned against the database release 13.8 (https://ftp.microbio.me/greengenes_release/gg_13_5/gg_13_8_otus.tar.gz). Sequences were quality-filtered, denoised, and merged, and chimeras were removed using the DADA2 plugin (23). Amplicon sequence variant (ASV) sequences were subsequently classified using the DADA2 and SILVA database (https://www.arb-silva.de/) to identify bacteria. Finally, the ASV table, including all samples, was summarized at various levels of taxonomic hierarchical structure (Table S2).

### Root metabolome analysis based on UPLC-Q-TOF/MS

Metabolome analysis was conducted as follows: plant tissues (0.2 g) were quickly frozen in liquid nitrogen immediately and ground into fine powder with a mortar and pestle. An aliquot of the powder (80 mg) was mixed with 0.8–1.6 mL pre-chilled methanol/acetonitrile/H_2_O (2:2:1, vol/vol) and vortexed thoroughly. The mixture was sonicated under low-temperature conditions for 30 min, followed by incubation at −20°C for 10 min. The sample was subsequently centrifuged at 14,000 × *g* for 20 min at 4°C. The supernatant was harvested and dried under vacuum. Prior to mass spectrometry (MS) analysis, the dried residue was reconstituted in 100 μL of acetonitrile/H_2_O (1:1, vol/vol), vortexed, and centrifuged again at 14,000 × *g* for 15 min at 4°C. The resulting supernatant was collected and injected for ultraperformance liquid chromatography coupled with quadrupole time-of-flight mass spectrometry (UPLC-Q-TOF/MS) analysis. To monitor the stability and repeatability of instrument analysis, quality control (QC) samples were prepared by pooling 10 μL of each sample and analyzed together with the other samples. QC samples were analyzed every four samples.

Analyses were performed using a UPLC (1290 Infinity LC, Agilent Technologies) coupled to a quadrupole time-of-flight (AB Sciex TripleTOF 6600; SCIEX, MA, USA) at Shanghai Personal Biotechnology Co., Ltd. (Shanghai, China). Samples were analyzed using a 2.1 mm × 100 mm ACQUIY UPLC BEH 1.7 μm HILIC column (Waters, Ireland). In both ESI-positive and -negative modes, the mobile phase contained A (25 mM ammonium acetate and 25 mM ammonium hydroxide in water) and B (acetonitrile). The gradient was 85% B for 1 min and was linearly reduced to 65% in 11 min, then was reduced to 40% in 0.1 min and kept for 4 min, and then increased to 85% in 0.1 min, with a 5 min re-equilibration period employed.

The ESI source conditions were set as follows: Ion Source Gas1 (Gas1) as 60, Ion Source Gas2 (Gas2) as 60, curtain gas (CUR) as 30, source temperature as 600°C, and Ion spray voltage floating (ISVF) as ±5,500 V. In MS-only acquisition, the instrument was set to acquire over the *m*/*z* range 60–1,000 Da, and the accumulation time for TOF MS scan was set at 0.20 s/spectrum. In auto-MS/MS acquisition, the instrument was set to acquire over the *m*/*z* range 25–1,000 Da, and the accumulation time for product ion scan was set at 0.05 s/spectra. The product ion scan is acquired using data-dependent acquisition (DDA), with high sensitivity mode selected. The parameters were set as follows: the collision energy (CE) was fixed at 35 V with ±15 eV; declustering potential (DP), 60 V (+), and −60 V (−); exclude isotopes within 4 Da, candidate ions to monitor per cycle of 10.

Raw MS data (wiff.scan files) were converted to MzXML files using ProteoWizard MSConvert before importing into freely available XCMS software. For peak picking, the following parameters were used: centWave *m*/*z* = 25 ppm, peakwidth = c (10, 60), and prefilter = c (10, 100). For peak grouping, bw = 5, mzwid = 0.025, and minfrac = 0.5 were used. Collection of Algorithms of MEtabolite pRofile Annotation (CAMERA) was used for the annotation of isotopes and adducts. In the extracted ion features, only the variables having more than 50% of the nonzero measurement values in at least one group were kept. Compound identification of metabolites was performed by comparing the accuracy of the *m*/*z* value (<25 ppm). Untargeted metabolomics analysis was performed without isotope internal standards for absolute quantification; pooled QC samples were used for signal normalization and batch effect correction. In-house databases of authentic standards and public databases, including KEGG, HMDB, MoNA, MassBank, METLIN, and NIST, were adopted for MS/MS spectral library searching. Metabolite structural annotation for biological samples was performed by matching multiple characteristic parameters, including retention time, molecular mass (mass tolerance: <25 ppm), MS/MS fragmentation patterns, and collision energy, with the data recorded in reference data from the above databases. All annotation results were further subjected to strict manual verification. Only metabolites annotated at Level 2 and above were retained for subsequent analysis. Duplicate metabolites detected in both ESI-positive and -negative modes were removed to avoid redundancy.

### Statistical analyses

Plant physiological assays were performed with four independent biological replicates. The significance of differences between the groups was determined through one-way analysis of variance (ANOVA) with Duncan’s test using SPSS V17.0. The 16S rRNA gene sequencing data were analyzed using R v.4.4.1. Shannon and Simpson indices were calculated using the “picante” and “vegan” packages, *P* value was obtained from the Kruskal-Wallis test, and intergroup differences were further assessed using Duncan’s test. Bray-Curtis principal coordinate analysis (PCoA) and linear discriminant analysis (LDA) effect size (LEfSe) analysis were both conducted on the Wekemo Bioincloud platform (https://www.bioincloud.tech). Permutational multivariate analysis of variance (PERMANOVA) was used to evaluate differences in microbial community composition based on Bray-Curtis PCoA, and ASVs with LDA >3.5 were selected as biomarkers for subsequent correlation analysis. A Spearman correlation clustering heatmap of biomarker ASVs was conducted, and Spearman correlation analysis between physiological index-associated ASVs and key metabolites was performed via the Personal Genescloud platform (https://www.genescloud.cn/home).

For metabolomic analysis, the processed data were analyzed by the R package (ropls), subjected to multivariate data analysis, including Pareto-scaled principal component analysis (PCA) and orthogonal partial least-squares discriminant analysis (OPLS-DA). UV normalization was applied to data sets prior to OPLS-DA and clustering heatmap analysis. Sevenfold cross-validation and response permutation testing were used to evaluate the robustness of the model. The variable importance in the projection (VIP) value of each variable in the OPLS-DA model was calculated to indicate its contribution to the sample classification. Metabolites with a VIP >1 were further applied to Student’s *t*-test at the univariate level to assess their statistical significance, with a *P* < 0.05 considered statistically significant. The OPLS-DA-based multiple-group volcano plot was obtained using the “ggplot2’ package with Benjamini-Hochberg (BH)-adjusted *P* values, while KEGG enrichment analysis was performed using the “tidyverse” package. K-means clustering analysis was conducted using an online Tutool platform (http://cloudtutu.com.cn/). Redundancy analysis (RDA) and Mantel tests between key microbial taxa and the metabolites selected by Pearson correlation heatmap were performed using the online platform of Shanghai Personal Biotechnology Co., Ltd. (https://www.genescloud.cn/home).

## RESULTS

### Divergent adaptation to salt stress across poplar varieties

To evaluate the salt tolerance of different poplar varieties, plantlets were cultured in coastal saline soil with high salinity (0.42%; a lethal concentration for the tested varieties), and the survival rate was calculated at 50 and 120 dpt. Throughout the experimental period, SXY exhibited the highest survival rate, while NL895 showed the lowest (Fig. S2).

**Fig 1:**
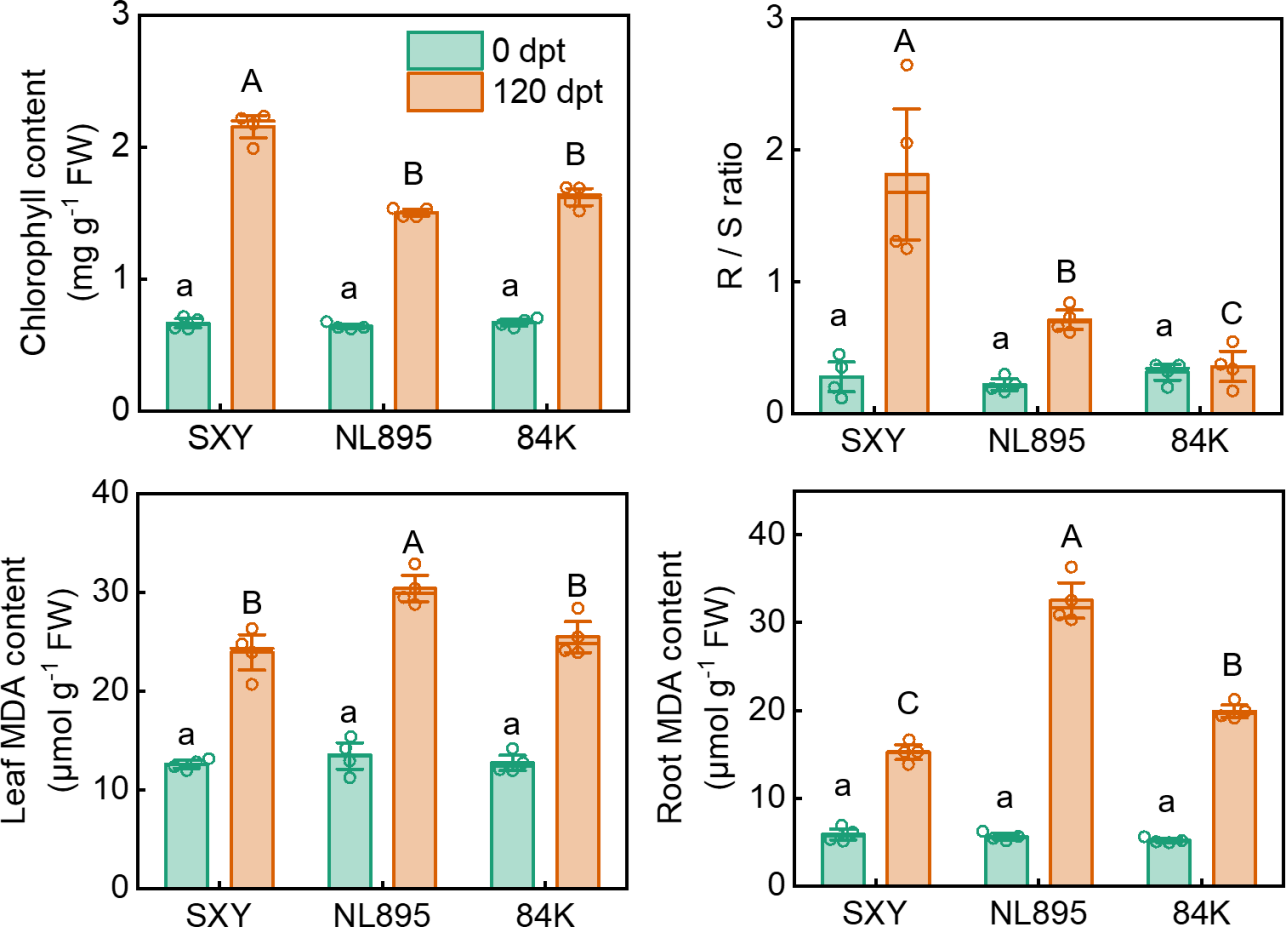
Effect of salt stress on chlorophyll content, membrane integrity, and growth of poplar varieties. R/S ratio, root/shoot ratio; MDA, malondialdehyde. Each data bar represents the mean index (mean ± SD). *n* = 4 plantlets in R/S ratio calculation; *n* = 4 biologically independent samples in determination assays of chlorophyll and MDA content; and lowercase letters represent significant differences among poplar varieties at 0 day post-transplanting (dpt), while uppercase letters indicate that at 120 dpt (*P* < 0.05; ANOVA, Duncan’s test).

In another set of culture experiments using soil with low salinity (0.27%; a non-lethal level), chlorophyll contents and MDA levels in leaves and roots, which indicate salt-induced damages, were determined at 0 and 120 dpt. Additionally, biomasses (characterizing plant growth) and phenotypes were assessed at 120 dpt. No discernible differences in damage indices (including chlorophyll and MDA levels) were observed among poplar varieties at 0 dpt, indicating uniform initial baseline values across all varieties. At 120 dpt, SXY exhibited the highest chlorophyll level and the lowest leaf and root MDA contents; NL895 displayed the lowest chlorophyll level and the highest MDA contents in both tissues; and 84K showed a chlorophyll level similar to that of NL895 but lower MDA contents in leaves and roots (Fig. 1 and Fig. S3A). Thus, NL895 suffered the most severe salt-induced injury, while SXY sustained the least damage.

With respect to growth parameters, 84K exhibited the highest shoot and root fresh weights prior to transplanting, while NL895 showed the lowest; this resulted in uniform R/S ratios among the tested poplar varieties. At 120 dpt, 84K had the highest shoot biomass, whereas SXY displayed the lowest. By contrast, SXY had higher root fresh weight than both NL895 and 84K, leading to the highest R/S ratio in SXY and the lowest in 84K (Fig. 1 and Fig. S3). These findings indicate that SXY exhibits the greater root growth under salt stress, while 84K preferentially maintains shoot growth. Collectively, the salt tolerance ranking of the tested poplar genotypes is SXY >84K >NL895.

### Rhizobacterial community composition of poplar in saline soil

**Fig 2:**
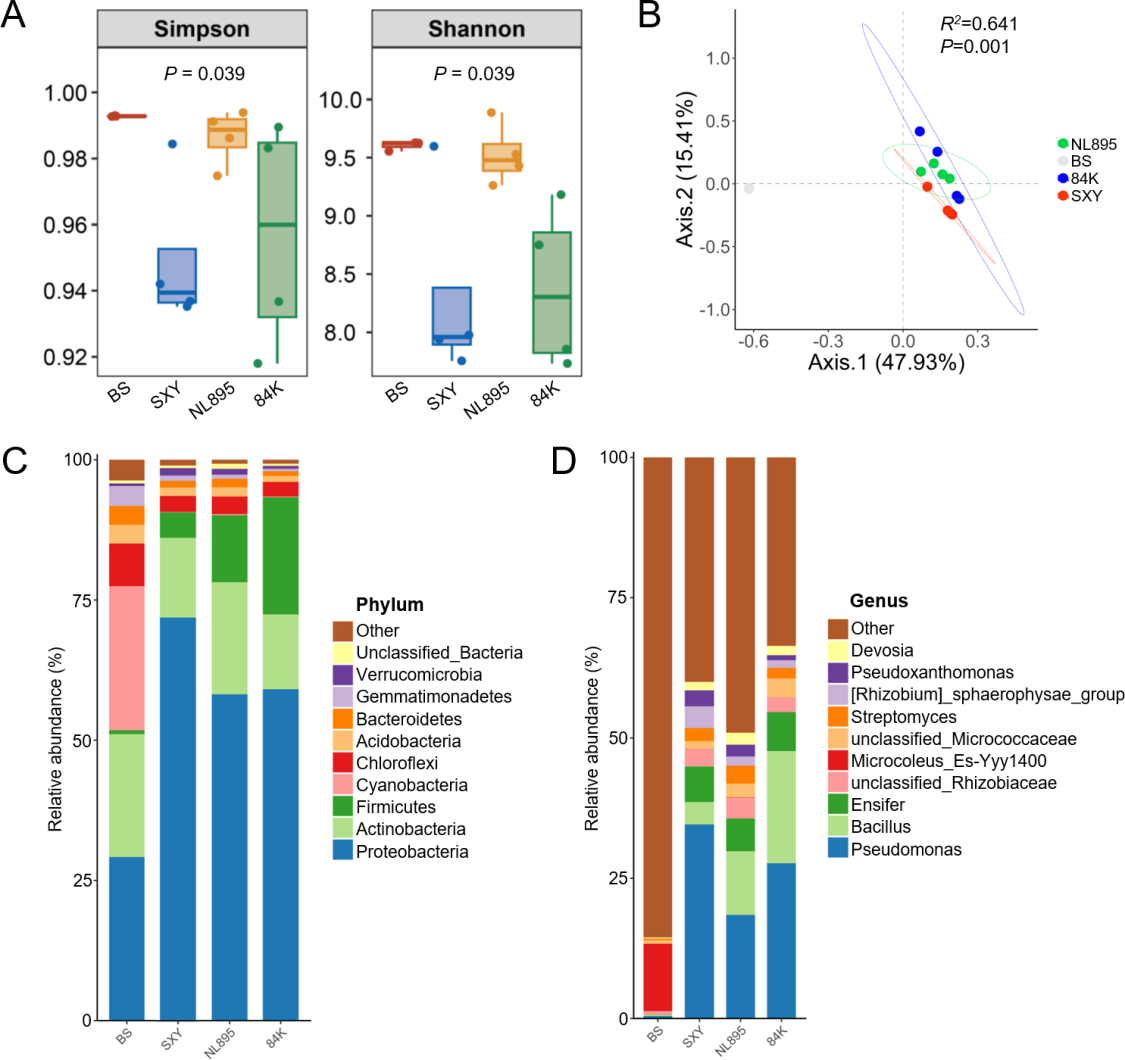
Microbial community composition of poplar varieties under salt stress. (**A**) α-diversity for the rhizosphere bacterial community of poplar varieties, and the *P*-value was calculated using ANOVA and Duncan’s test. BS, bulk soil. (**B**) PCoA based on the Bray-Curtis dissimilarity (ASV level) for the rhizosphere bacterial community (*P* = 0.001; one-way PERMANOVA). Relative abundance of the top 10 most abundant (**C**) phyla and (**D**) genera of microbial community from poplar under salt stress. Samples from the BS and the rhizosphere of poplar were collected at 120 dpt.

A total of 1,293,298 classifiable 16S rRNA sequence reads were generated from poplar rhizosphere and bulk soil (BS; Table S2) samples. The mean number of non-singleton sequences per sample was 43,661 (Table S3). Compared to BS, Simpson and Shannon indices of rhizobacterial communities in SXY and 84K significantly decreased, with SXY exhibiting the lowest for both indices (Fig. 2A). This finding implies the enrichment of specific bacterial taxa in the rhizosphere of SXY and 84K under salt stress. Principal coordinate analysis (PCoA) based on Bray-Curtis dissimilarity unveiled significant differences in bacterial community composition between rhizosphere soil and BS along axis 1. Specifically, the rhizobacterial community of SXY was separated from the other varieties along axis 2, while discernible divergences in the rhizobacterial composition were also observed between NL895 and 84K (Fig. 2B).

We next investigated the top 10 most abundant phyla and found that compared to BS, Proteobacteria, Firmicutes, and Verrucomicrobia were enriched, whereas the abundances of Actinobacteria, Cyanobacteria, Chloroflexi, Acidobacteria, Bacteroidetes, and Gemmatimonadetes were reduced in the rhizosphere of all poplar varieties under salt stress. Across all varieties, SXY exhibited the greatest enrichment of Proteobacteria and Verrucomicrobia, while 84K showed the highest enrichment of Firmicutes (Fig. 2C and Table S4). Among the top 10 genera, all taxa except *Microcoleus_Es-Yyy1400* were enriched in the poplar rhizosphere compared to BS under salt stress. Specifically, *Pseudomonas*, [*Rhizobium*]_sphaerophysae_group, and *Pseudoxanthomonas* were most highly enriched in SXY, *Bacillus* in 84K, and *Streptomyces* in NL895 (Fig. 2D and Table S5).

**Fig 3:**
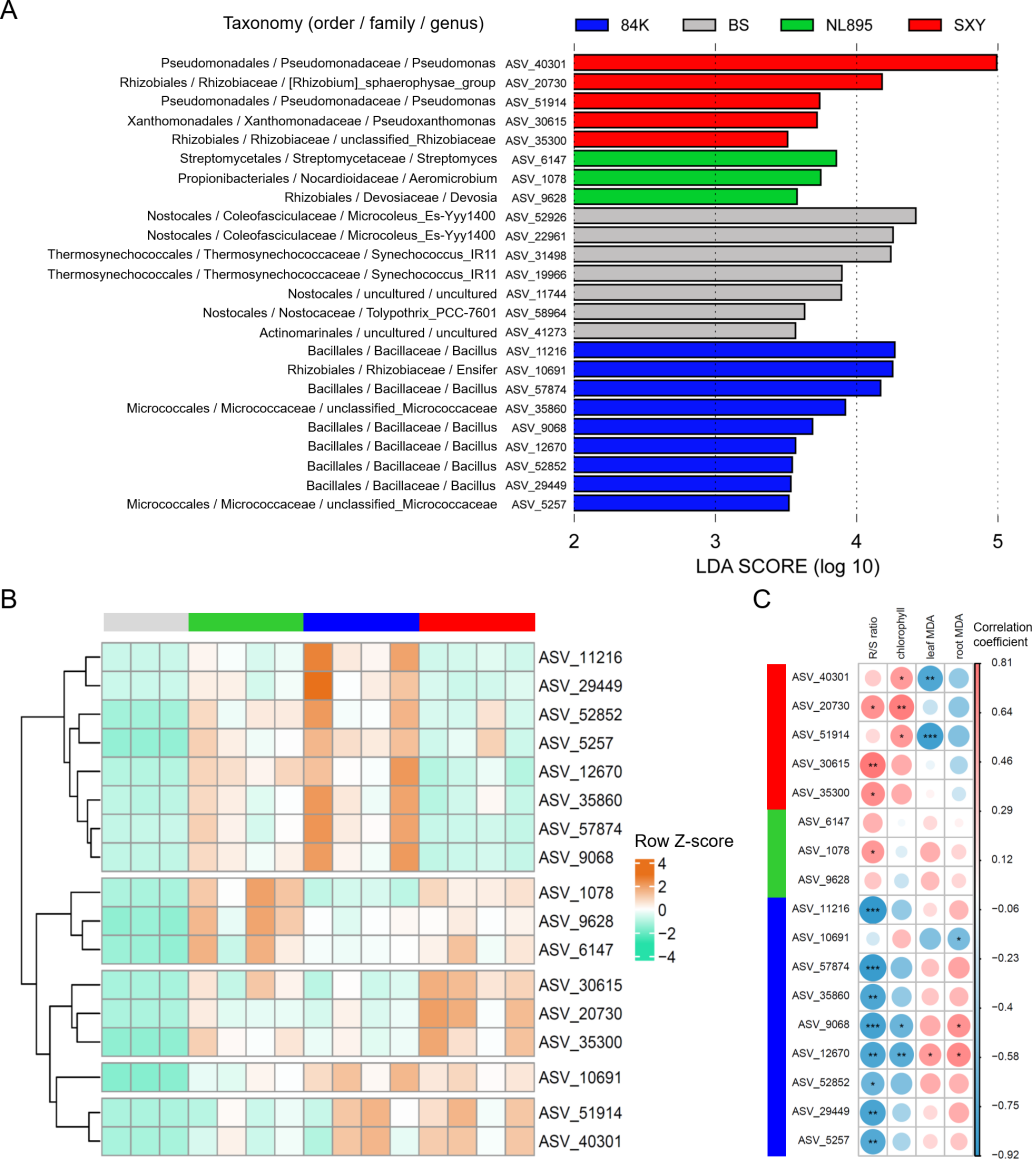
Salt tolerance-associated microbial groups in the poplar rhizosphere. (**A**) Biomarkers at the ASV-level of poplar varieties identified by LefSe analysis (LDA > 3.5). (**B**) Clustering heatmap based on Spearman correlation analysis for the abundance of poplar-enriched ASVs. Each clustering heatmap is color-coded based on normalized Z-scores calculated from the relative abundance of the labeled ASV. (**C**) Spearman correlation between the enriched ASVs and physiological indices; the color scale indicates correlation coefficient; and asterisks indicate significance of differences (asymptotic *t*-test; *P < 0.05, **P < 0.01, and ***P < 0.001).

To further identify poplar-enriched rhizobacterial taxa under salt stress, we performed linear discriminant analysis (LDA) effect size (LEfSe) analysis at the ASV level (LDA >3.5). Our results indicated that the biomarkers of rhizobacterial community in SXY primarily belonged to Rhizobiaceae (40%), *Pseudomonas* (40% ASVs), and *Pseudoxanthomonas* (20%); those of the 84K rhizosphere mainly comprised ASVs assigned to *Bacillus* (66.7%), Micrococcaceae (22.2%), and *Ensifer* (11.1%); and NL895 harbored three distinct biomarkers that came from *Streptomyces*, *Aeromicrobium,* and *Devosia*, respectively (Fig. 3A). A clustering heatmap grouped these ASVs into five clusters, revealing that the 84K-enriched ASVs showed similar abundance patterns—with the highest abundances in 84K and the lowest in SXY, with the exception of ASV_10691 (assigned to *Ensifer*), which was more enriched in SXY than in NL895. Additionally, the NL895-specific biomarker ASVs exhibited the lowest abundances in SXY. The SXY-enriched ASVs were clustered into two groups: one comprised ASV_51914 and ASV_40301 (both assigned to *Pseudomonas*), which also exhibited higher abundance in the 84K rhizosphere than those in NL895; the other included the remaining ASVs, which were uniquely abundant in the SXY rhizosphere (Fig. 3B).

To investigate the association between rhizobacteria recruited by different poplar varieties and host salt tolerance, we performed Spearman correlation analysis between biomarkers and phenotypic parameters at 120 dpt, including growth indicator R/S ratio and damage indices such as chlorophyll content and MDA levels (Fig. 3C). Biomarkers of SXY were either positively correlated with R/S ratio and chlorophyll content or negatively correlated with leaf and root MDA levels. ASVs from *Pseudomonas* showed a positive correlation with chlorophyll content and a negative correlation with leaf MDA level; the Rhizobiaceae ASV_20730 exhibited a positive correlation with both R/S ratio and chlorophyll content; and ASVs belonging to *Pseudoxanthomonas* were mainly positively correlated with R/S ratio. With respect to biomarkers of NL895, only ASV_1078 (assigned to *Aeromicrobium*) showed a discernible positive correlation with R/S ratio. Most 84K-enriched ASVs exhibited an opposite correlation pattern to those of SXY, except for the *Ensifer* ASV_10691, which was negatively correlated with root MDA level. Taken together, these results demonstrate that poplar varieties assemble distinct rhizobacterial communities in a host genotype-dependent manner under salt stress, and the most salt-tolerant poplar genotype possesses enriched specific rhizobacterial taxa from *Pseudomonas*, *Pseudoxanthomonas*, and Rhizobiaceae that are associated with host salt adaptation.

### Metabolome profiles of roots from poplar varieties under salt stress

**Fig 4:**
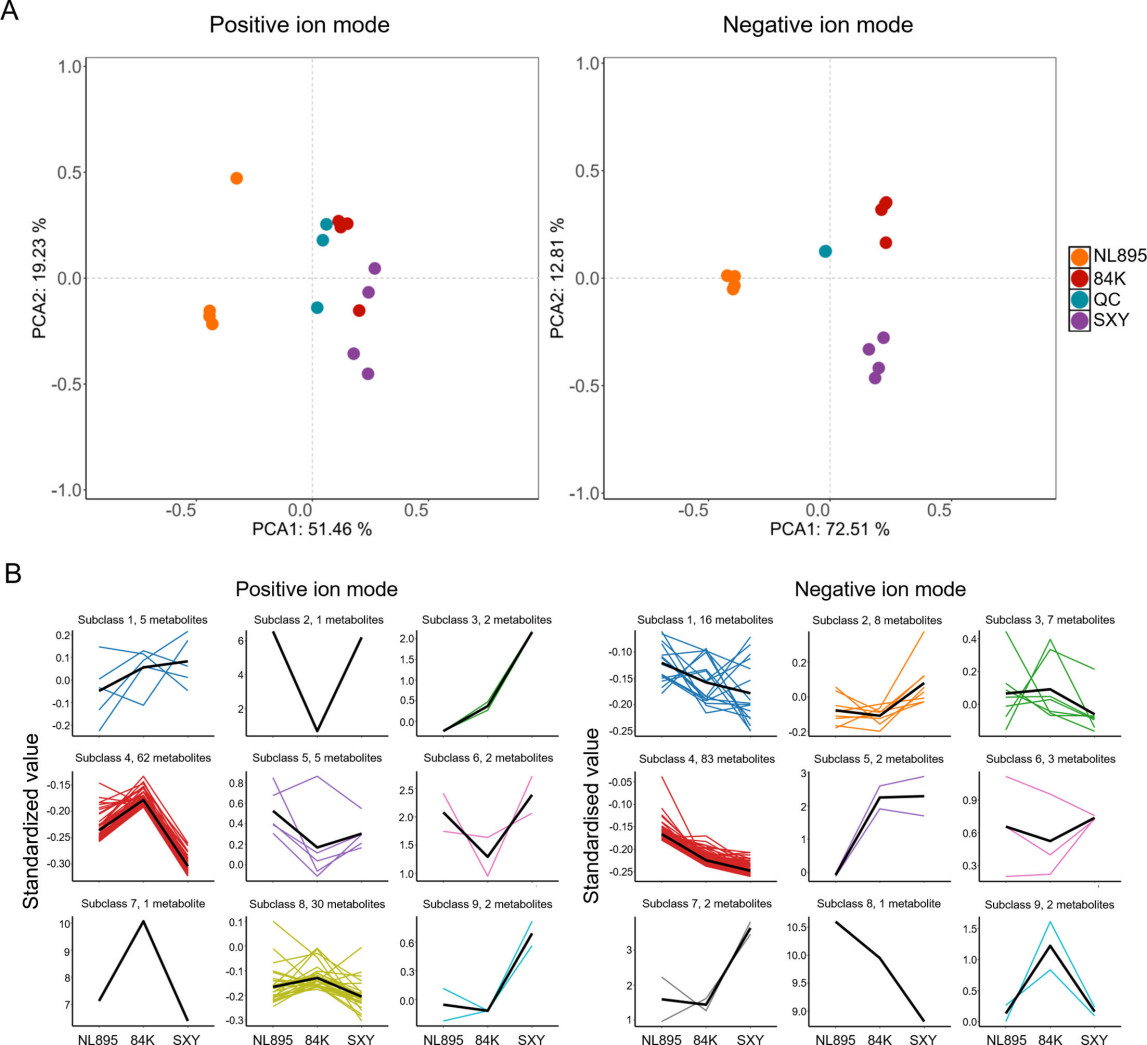
Metabolic profiles in roots of poplar varieties under salt stress. (**A**) PCA of metabolic profiles in roots of poplar varieties. QC, quality control. (**B**) K-means clustering of the annotated metabolites from roots of poplar varieties under positive and negative ion mode.

To explore the root metabolic patterns of poplar varieties under salt stress, we performed untargeted metabolomic profiling of poplar roots using a UPLC-Q-TOF/MS platform. Root samples corresponding to the rhizosphere soils used in the above bacterial profiling were analyzed (12 samples in total), and 236 metabolites were annotated (111 under positive ion mode and 125 under negative mode; Table S6). Principal component analysis (PCA) revealed distinct metabolomic profiles with clear separation among the tested poplar varieties in both positive and negative ion modes. In detail, NL895 was separated from the other varieties along axis 1 in both ion modes. Separation along axis 2 was observed between SXY and 84K in both ion modes, which was more pronounced in the negative ion mode (Fig. 4A). These results indicate that the root metabolome profiles of poplar varieties under salt stress are genotype-dependent.

To characterize divergences in root metabolic profiles among poplar varieties, we identified differentially accumulated metabolites (DAMs; |log_2_fold change| > 1, VIP >1, and BH-adjusted *P* < 0.05) based on orthogonal partial least squares discriminant analysis (OPLS-DA). Combining data from positive and negative ion modes, we identified 49 DAMs in the NL895 vs SXY comparison group, 51 in NL895 vs. 84K and 41 in SXY vs. 84K (Fig. S4 and Table S7). These results indicate greater similarity in metabolic patterns between SXY and 84K, which is consistent with our PCA results (Fig. 4A). Based on these DAMs, we conducted KEGG enrichment analysis. Considering that “ferroptosis,” “vascular smooth muscle contraction,” and “vitamin digestion and absorption” are pathways that occur in animals and humans, we found that, compared to other varieties, NL895 mainly enriched lipid metabolism-related pathways, such as “alpha‌-linolenic acid metabolism” and “biosynthesis of unsaturated fatty acids.” SXY exhibited more active xenobiotics biodegradation and metabolic pathways, especially “aminobenzoate degradation” and “benzoate degradation,” which belong to secondary metabolism, especially in the comparison of NL895 vs. SXY. Moreover, 84K showed enriched secondary metabolic pathways, including “biosynthesis of phenylpropanoids,” “biosynthesis of secondary metabolites,” “flavonoid biosynthesis,” and “biosynthesis of alkaloids derived from shikimate pathway,” as well as the membrane transport pathway “ABC transporters,” and “microbial metabolism in diverse environments” (Fig. S5 and Table S9). Although not all of these pathways showed significant differences between varieties, the activity of secondary metabolism was, to a certain extent, associated with plant salt tolerance.

To investigate the metabolites most highly enriched in roots of the most salt-tolerant variety SXY, we performed K-means clustering analysis. All 236 annotated metabolites from the NL895 vs. SXY, NL895 vs. 84K, and SXY vs. 84K comparisons were classified into nine clusters in positive and negative ion modes, respectively (Fig. 4B). Subclasses 1, 3, 6, and 9 in positive ion mode corresponded to metabolites with the highest accumulation in SXY roots, while subclasses 2, 5, 6, and 7 in negative ion mode showed the same pattern. Subsequently, metabolites from these subclasses (26 compounds in total) were listed in Table S8. Specifically, secondary metabolites accounted for the largest proportion (38.5%), mainly comprising phenolic acids, flavonoids, triterpenoids, and their derivatives. Fatty acids and conjugates represented 30.8% of the total compounds, and phospholipids (e.g., glycerophosphocholines, monoalkyl phosphates, and phosphocholines) accounted for 15.4%. The remaining compounds were classified as carbohydrates and carbohydrate conjugates. These results further support the conclusion that SXY exhibits high levels of specific secondary metabolites. Collectively, poplar varieties display genotype-specific metabolic patterns under salt stress.

### Interaction between rhizobacterial taxa and root metabolism in poplar salt tolerance

**Fig 5:**
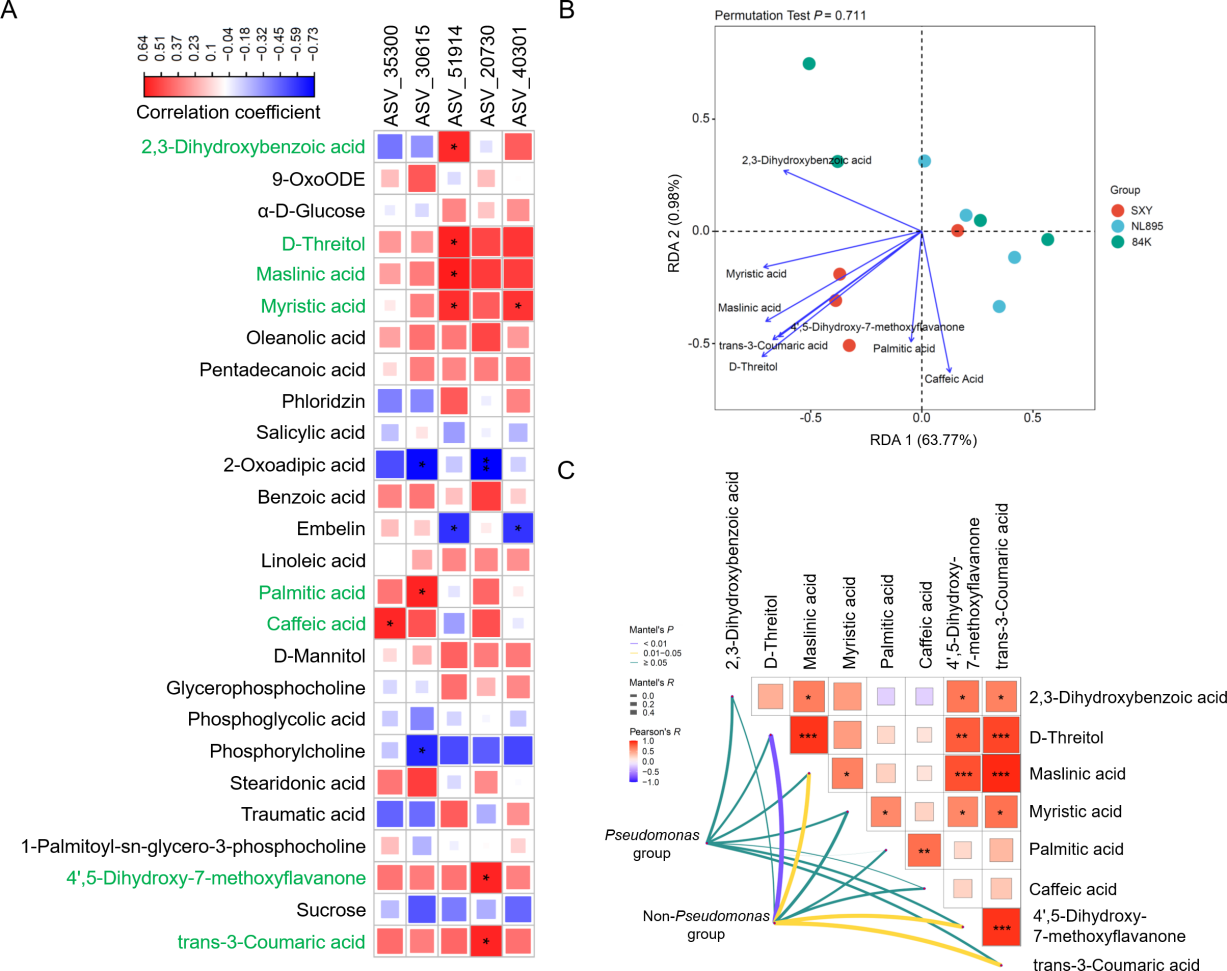
Interactions between root metabolites and key microbial taxa in poplar under salt stress. (**A**) Spearman correlation between the bacterial ASVs and metabolites that both are enriched in the most salt-tolerant poplar variety. The color scale represents correlation coefficient; and asterisks indicate significance of differences (asymptotic *t*-test; *P < 0.05). The green compounds represent metabolites with significantly positive correlation with at least one ASV enriched by the salt-tolerant poplar. (**B**) Redundancy analysis (RDA) ordination plot illustrating the association between the root metabolites and the community structure of key microbial taxa across three poplar varieties. The contents of compounds were used as explanatory variables. PERMANOVA was performed to assess the significance of the RDA models. (**C**) Mantel tests between key microbial taxa and metabolites. *Pseudomonas* matrix includes ASV_40301 and 51914; Non-*Pseudomonas* matrix is composed of ASV_20730, 30615, and 35300. Mantel tests were performed using the weighted UniFrac (WUF) distance matrix and the Euclidean distance matrix of the contents of each metabolite. The color scale represents Pearson’s correlation coefficient, and asterisks show significance of differences (asymptotic *t*-test; *P < 0.05, **P < 0.01, and ***P < 0.001).

To identify metabolites involved in interactions with rhizobacteria that influence plant salt tolerance, we performed Spearman correlation analysis between metabolites highly enriched in SXY roots and rhizobacterial taxa associated with host salt tolerance. We identified eight metabolites showing positive correlations with salt tolerance-associated ASVs, including five secondary metabolites, two long-chain fatty acids, and one carbohydrate derivative. To be specific, these included myristic acid, which was positively correlated with ASV_40301 and ASV_51914; 4′,5-dihydroxy-7-methoxyflavanone and trans-3-coumaric acid with ASV_20730; 2,3-dihydroxybenzoic acid, D-threitol, and maslinic acid with ASV_51914; palmitic acid with ASV_30615; and caffeic acid with ASV_35300 (Fig. 5A). Collectively, the most salt-tolerant poplar genotype accumulates high levels of secondary metabolites, long-chain fatty acids, and a carbohydrate derivative, which potentially interact with bacterial taxa from *Pseudomonas*, *Pseudoxanthomonas,* and Rhizobiaceae under salt stress.

To explore whether the above eight compounds drive the community variation of key microbial taxa across poplar varieties under salt stress, we performed forward RDA using the levels of these eight metabolites as explanatory variables. We found that the concentrations of these compounds collectively explained 64.75% of the microbial community variation (axes 1 and 2 accounted for 63.77% and 0.98%, respectively; Fig. 5B; *P* = 0.711, PERMANOVA). Moreover, these metabolites were associated with the abundance of key taxa in SXY under salt stress, with D-threitol, maslinic acid, 4′,5-dihydroxy-7-methoxyflavanone, and trans-3-coumaric acid exhibiting the strongest correlations. In a reverse RDA model using the abundance of the key microbial taxa as explanatory variables, the first two axes explained 56.81% of variation in the eight metabolites (Fig. S6 and *P* = 0.194, PERMANOVA), indicating the reciprocal regulation between metabolites and microbial taxa. However, the greater explanatory power of the forward model suggests that these compounds may drive the variation of key rhizosphere taxa in the most salt-tolerant poplar variety under salt stress.

To examine the correlation between eight candidate metabolites and the community variation of key microbial taxa under salt stress, we divided the five salt tolerance-related ASVs into two groups according to their abundance profiles under salt stress (Fig. 3B): the *Pseudomonas* group (ASV_40301 and ASV_51914) and the non-*Pseudomonas* group (ASV_20730, ASV_30615, and ASV_35300). Mantel tests were subsequently performed between the two microbial groups and the eight candidate metabolites. We observed a striking dichotomy in metabolite-microbe associations: the non-*Pseudomonas* group was significantly associated with four key metabolites (D-threitol, maslinic acid, 4′,5-dihydroxy-7-methoxyflavanone, and trans-3-coumaric acid; Mantel’s *R* > 0.2, Mantel’s *P* < 0.05), whereas the *Pseudomonas* group showed no significant associations with most of these metabolites (Fig. 5C and Table S10). These findings corroborate our RDA results. Thus, poplar roots may drive variation in the rhizosphere microbial community mainly through the combined effects of D-threitol, maslinic acid, 4′,5-dihydroxy-7-methoxyflavanone, and trans-3-coumaric acid on the non-*Pseudomonas* group under salt stress.

## DISCUSSION

Plants with different genetic backgrounds exhibit divergent stress tolerance, which is associated with their specific root microbial communities under stress (10, 16, 24). Here, we demonstrated that three poplar varieties showed differential salt tolerance, as evidenced by the divergent responses of damage parameters and growth performance to salt stress. Moreover, these poplar varieties formed distinct rhizosphere bacterial communities under salt stress with particular microbial biomarkers, consistent with previous studies (24, 25). This indicates that different poplar varieties adopt specific survival strategies involving plant-microbe interactions under salt stress. Through Spearman correlation analysis, we further found that rhizosphere *Pseudomonas*, *Pseudoxanthomonas*, and Rhizobiaceae taxa were strongly enriched in the most salt-tolerant variety SXY, which were correlated to host salt tolerance parameters. *Pseudomonas* and Rhizobiaceae are well characterized PGPRs that help hosts cope with stress (26–28), and *Pseudoxanthomonas* sp. was reported to improve plant salt tolerance by producing indoleacetic acid, proline, and exopolysaccharides (29). This may, at least partly, explain why SXY exhibits high salt tolerance. We also demonstrated that 84K enriched an *Ensifer* ASV that was negatively correlated with root MDA, which might enhance host salt adaptation by synthesizing stress-related phytohormones under salinity (30). Most 84K*-*enriched *Bacillus* taxa were negatively correlated with plant salt-tolerance traits, inconsistent with some previous studies (31). This might be because they did not directly affect poplar but participated in the host salt tolerance by interacting with other microbes or abiotic environmental factors (32, 33).

Plants coordinate metabolic reprogramming in response to abiotic stress in a genotype-dependent manner (34, 35). Using an untargeted metabolomic approach, we found that poplar varieties with different genetic backgrounds exhibited distinct metabolic patterns in roots under saline conditions, consistent with previous studies (36, 37). This may be attributed to divergent salt stress responses arising from distinct molecular mechanisms in plants of different genotypes. Furthermore, K-means clustering unveiled that the most salt-tolerant genotype SXY exhibited higher levels of 26 metabolites than other varieties, which mainly comprised secondary metabolites, along with several fatty acids, carbohydrates, and their conjugates. Previous studies underlined their function not only in plant endogenous tolerance, such as antioxidants (e.g., phenolic acids) (38), phytoalexins (4′,5-dihydroxy-7-methoxyflavanone) (39), and osmotic adjustment substances (fatty acids and carbohydrates) (40, 41) but also as signaling molecules for communication with soil beneficial microbes (42). Thus, the salt-tolerant poplar plants may alleviate salt stress by accumulating these metabolites to modulate their endogenous metabolism and/or interact with the plant microbiome.

Plants interact with the rhizosphere microbiome during adaptation to stress by modulating genotype-specific root metabolites (10, 16, 24). Using Spearman correlation analysis, we demonstrated significant associations between eight SXY-enriched metabolites—mainly secondary metabolites, along with several long-chain fatty acids and carbohydrate derivatives—and salt tolerance-related rhizobacteria (e.g., *Pseudomonas*, *Pseudoxanthomonas*, and Rhizobiaceae taxa). RDA unfolded that these metabolites, particularly four key metabolites including D-threitol, maslinic acid (a triterpene), 4′,5-dihydroxy-7-methoxyflavanone, and trans-3-coumaric acid, potentially drive variation of key rhizosphere microbial taxa in the most salt-tolerant poplar genotype under salt stress. Subsequent Mantel tests confirmed the correlations between the above four metabolites and key microbial taxa belonging to *Pseudoxanthomonas* and Rhizobiaceae. Together with the associations of D-threitol and maslinic acid with the *Pseudomonas* ASV_51914 in the Spearman heatmap, we found that the salt-tolerant poplar genotype may accumulate D-threitol, maslinic acid, 4′,5-dihydroxy-7-methoxyflavanone, and trans-3-coumaric acid to regulate key microbial taxa under salt stress. Previous studies showed that these four key compounds are common growth substances or ‌chemoattractants for specific beneficial microbes (10, 43), making them potential factors orchestrating the rhizosphere microbiome to handle environmental stresses (16, 44–46). This could explain the specific enrichment of rhizosphere *Pseudomonas*, *Pseudoxanthomonas*, and Rhizobiaceae taxa in the salt-tolerant poplar variety SXY.

Colonization of beneficial microbes in the rhizosphere can influence host root metabolism in the face of adverse environments (7, 11). Using key microbial taxa as explanatory variables, we found that the reverse RDA model had a slightly lower explanatory power (56.81%) for the eight candidate metabolites than the forward model (64.75%). Thus, the reciprocal regulation possibly exists: key microbial taxa may also influence the root metabolism through mechanisms like enzyme secretion, signal transduction, or activation of host tolerance (47). This might result in the accumulation of certain of the eight associated compounds (e.g., 2,3-dihydroxybenzoic acid or caffeic acid) (48), which, in turn, play protective roles in poplar salt tolerance. These findings provide deeper insights into plant resistance mechanisms.

Although associations were identified between root metabolites and key microbial taxa, the causal links remain inferential due to the lack of *in situ*-tracing experiments. Moreover, the molecular mechanisms underlying these beneficial interactions remain elusive, as genetic functional validation has not yet been conducted. To gain more direct evidence, future studies may employ an axenic co-culture system, microbial isolation and inoculation, and exogenous metabolite application, together with integration of transcriptome analysis and transgenic plant lines, to clarify how plant metabolism shapes the root microbiome under salt stress and the underlying molecular mechanisms.

**Fig 6:**
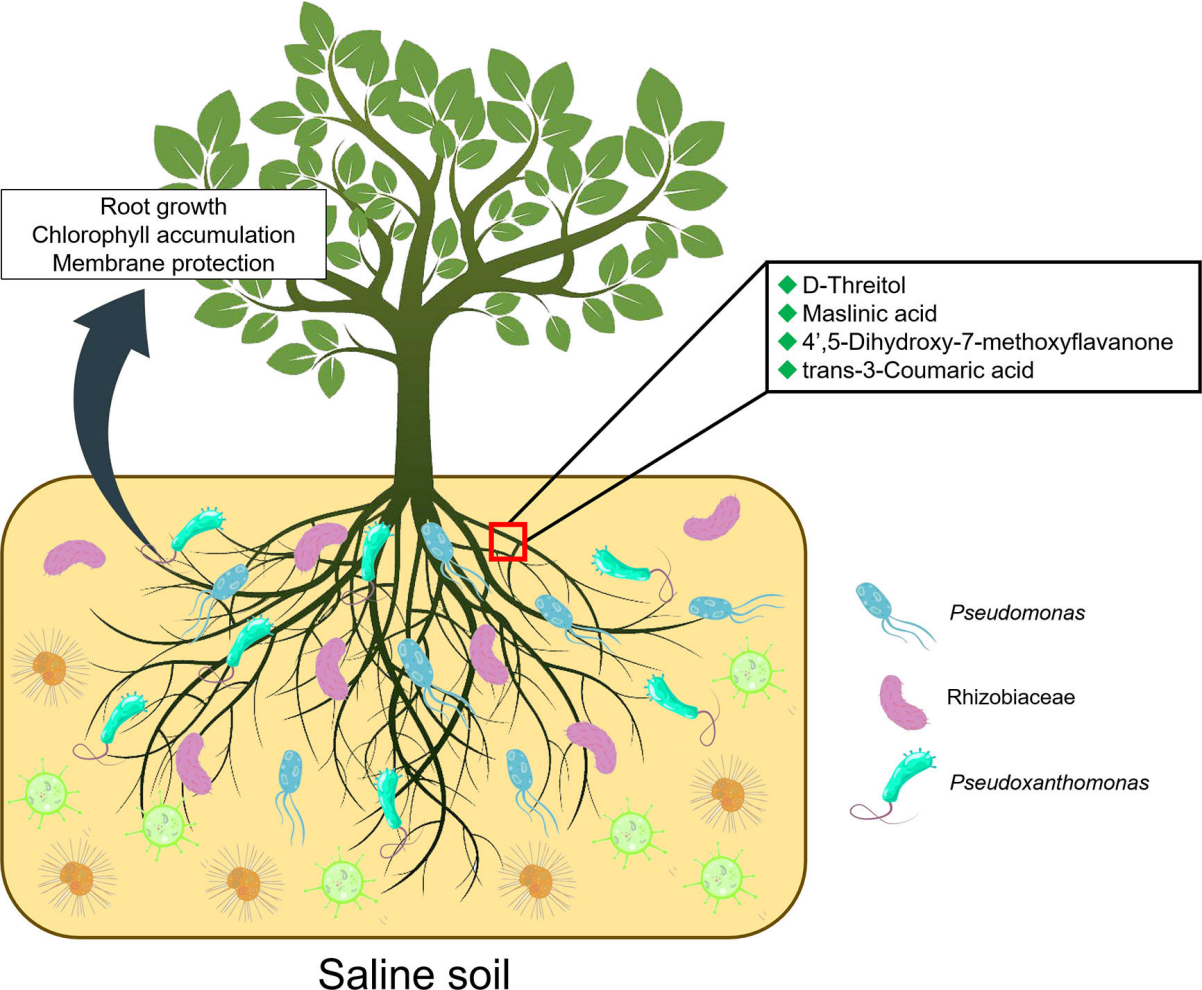
Proposed model of regulatory mechanism for the interaction between rhizobacterial community and root metabolism in poplar salt tolerance.

In conclusion, we showed that SXY exhibited higher salt tolerance than either NL895 or 84K. Under salt stress, SXY assembled a distinct rhizosphere microbial community in the rhizosphere with enriched specific salt tolerance-associated bacterial taxa from *Pseudomonas*, *Pseudoxanthomonas,* and Rhizobiaceae. SXY roots accumulated D-threitol, maslinic acid, 4′,5-dihydroxy-7-methoxyflavanone, and trans-3-coumaric acid, which may mediate the enrichment of the beneficial *Pseudomonas*, *Pseudoxanthomonas,* and Rhizobiaceae taxa in poplar. Based on these results, we demonstrate the potential interaction between rhizobacterial communities and root metabolism patterns and their effect on poplar salt tolerance (Fig. 6). Our findings provide new insights into the mechanisms by which poplar varieties harness soil microbiota to adapt to salt stress, offering a theoretical basis for innovation in microbial application methods in agroforestry.

## Data Availability

All raw plant RNA-seq data and rhizosphere bacterial 16S rRNA sequencing data reported in this paper were deposited in the Sequence Read Archive (http://www.ncbi.nlm.nih.gov/sra) under NCBI BioProject number PRJNA1305661. The metabolomics data supporting this study are openly accessible in the Metabolights database at https://www.ebi.ac.uk/metabolights/MTBLS14479.

## SUPPLEMENTAL MATERIAL

Supplemental figures msystems.00635-26-s0001.docx Fig. S1 to S6.

Supplemental tables msystems.00635-26-s0002.xlsx Tables S1 to S10.

ASM does not own the copyrights to Supplemental Material that may be linked to, or accessed through, an article. The authors have granted ASM a non-exclusive, world-wide license to publish the Supplemental Material files. Please contact the corresponding author directly for reuse.
