# Supplementary material for: Interaction between rhizobacterial community and host root metabolism influences poplar salt tolerance
Source: mSystems. 2026 Jun 17;11(7):e00635-26. doi: 10.1128/msystems.00635-26 (PMC13386973; doi:10.1128/msystems.00635-26)
Supplement: Supplemental figures — Fig. S1 to S6. [file msystems.00635-26-s0001.docx]

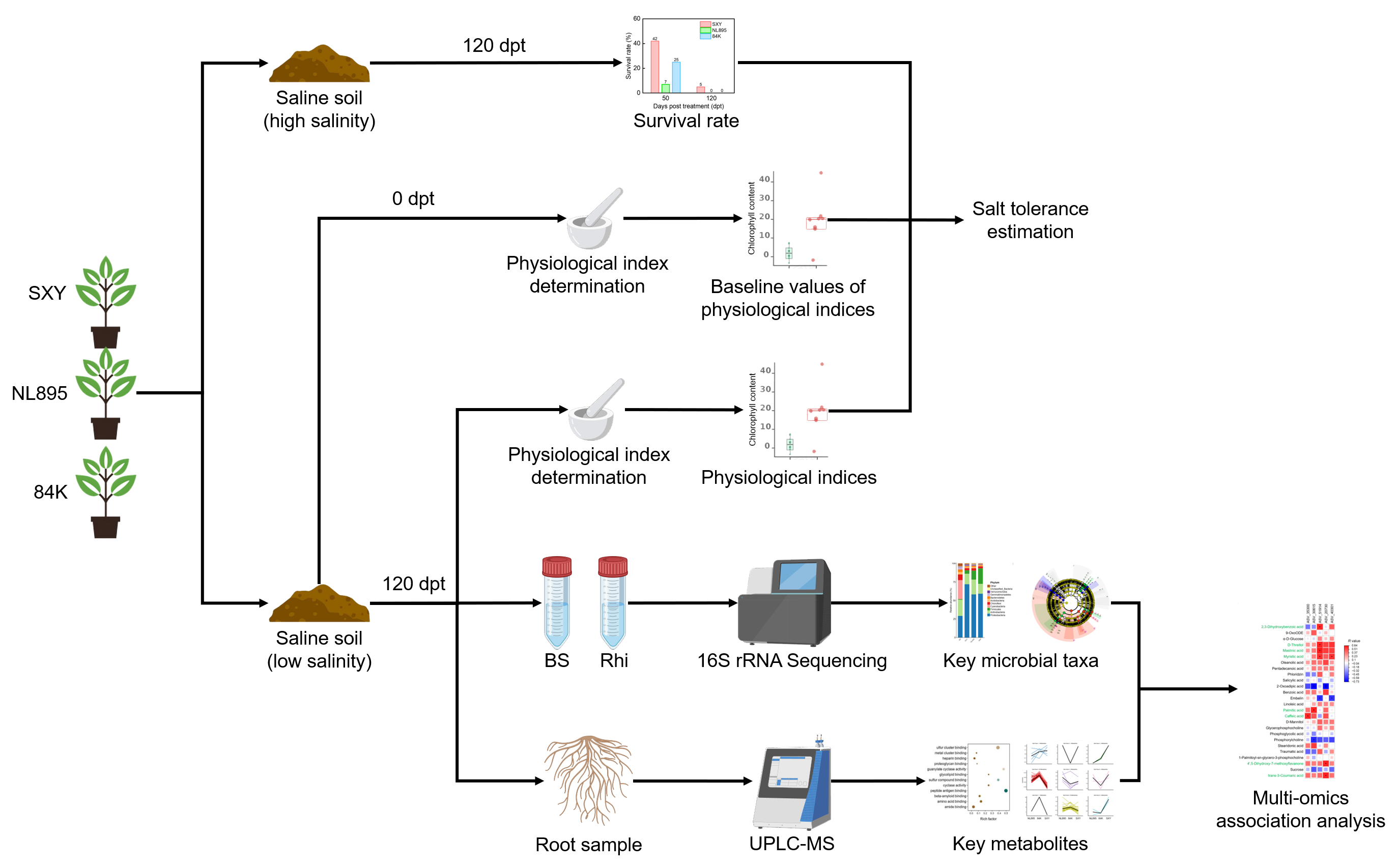
 **FIG S1** An overview of the experimental workflow for the present study. BS, bulk soil; Rhi, rhizosphere soil; dpt, days post transplanting.


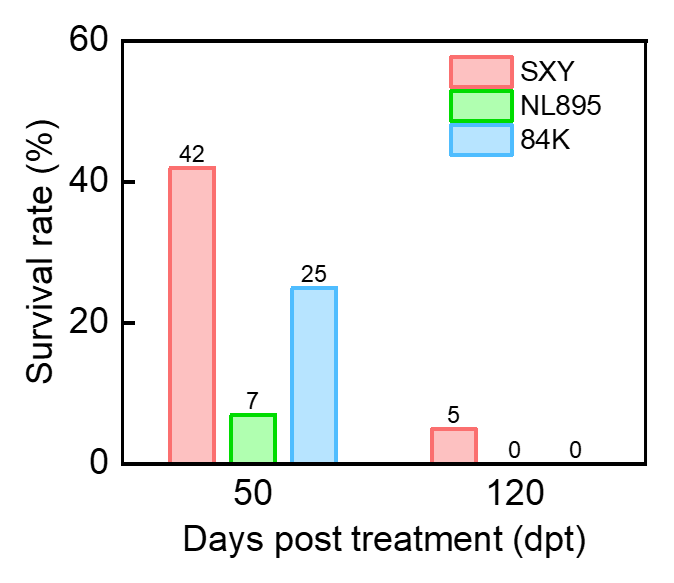
**FIG S2** Survival rate of poplar varieties cultured under high salinity.


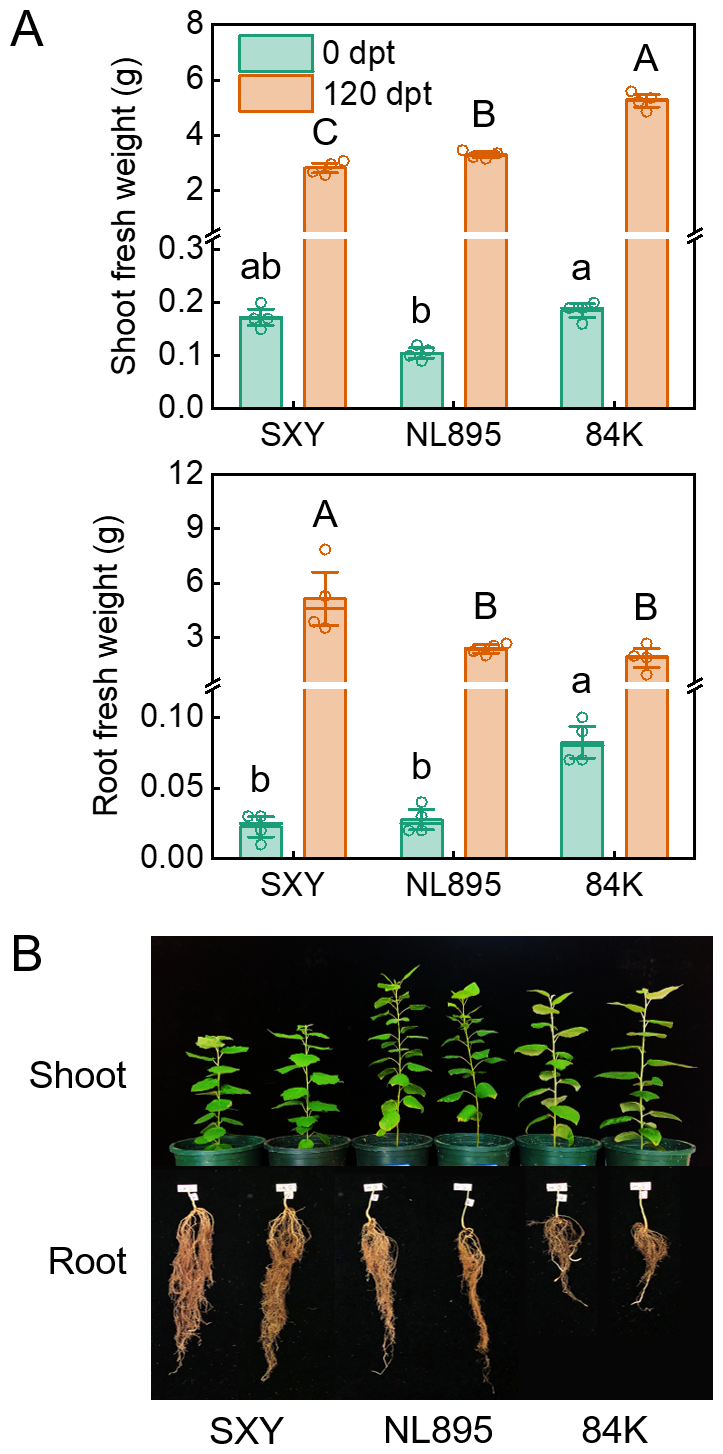
**FIG S3** Effect of low salinity on poplar varieties. (A) Biomass of poplar varieties at 0 and 120 dpt. Each data bar in represents the mean index (mean ± SD). n = 4 plantlets in biomass measurement; lowercase letters represent significant differences among poplar varieties at 0 dpt while uppercase letters indicates that at 120 dpt (*P* < 0.05; ANOVA, Duncan’s test). (B) Phenotypes of three poplar varieties at 120 dpt.


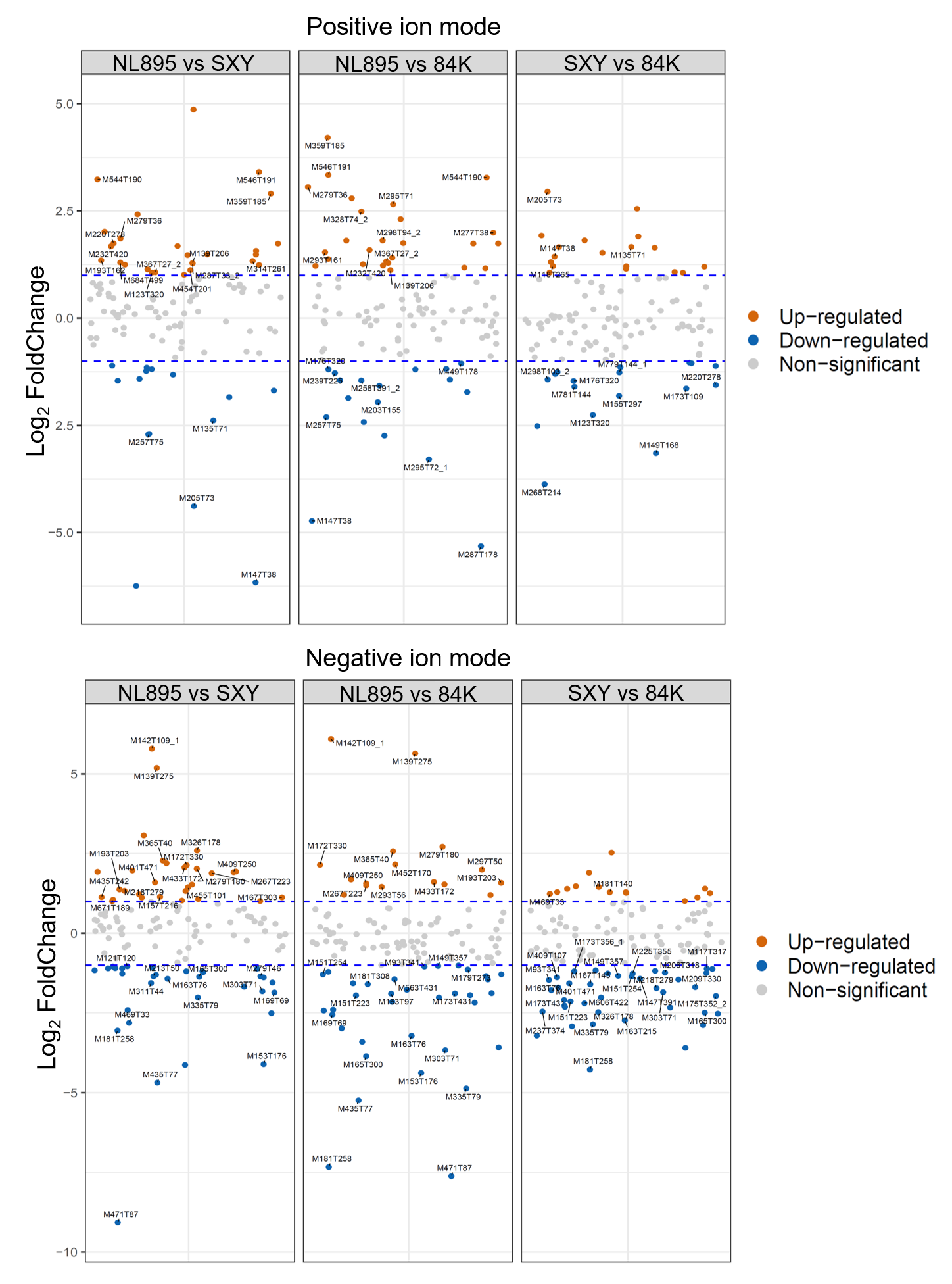
**FIG S4** Differentially accumulated metabolites (DAMs) between poplar varieties based on orthogonal partial least squares discriminant analysis (OPLS−DA). The group after “vs” stands for “control”. DAMs with |log2FoldChange| >1, VIP >1, and *P* <0.05 (BH−adjusted) were labeled with compound ID.


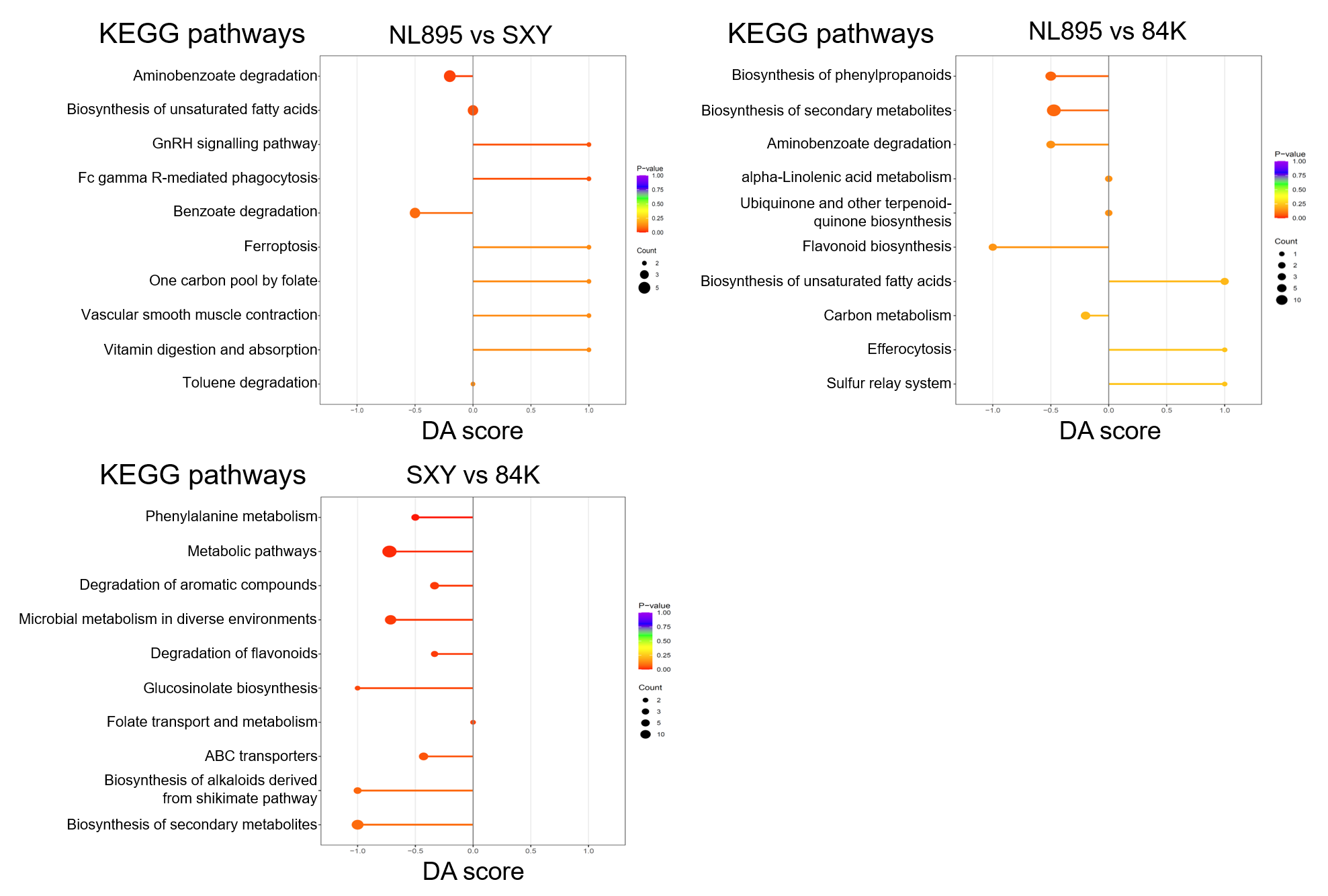
**FIG S5** KEGG enrichment of DAMs in each comparison. The orientation of each horizontal line denotes the regulatory trend of DAMs: the dot at the line’s left terminus corresponds to down-regulated DAMs, while the right terminus stands for up-regulated ones. The vertical axis lists the names of all differential metabolic pathways. The horizontal axis shows the differential abundance score (DA score), which reflects the overall metabolic variation in each pathway. Positive values indicate up-regulation of metabolites, whereas negative values indicate down-regulation. The length of each horizontal segment corresponds to the absolute DA score; the diameter of the circular dots at each segment’s endpoints represents the number of DAMs associated with the pathway; and the color gradient of both segments and dots indicates the magnitude of *P* value.


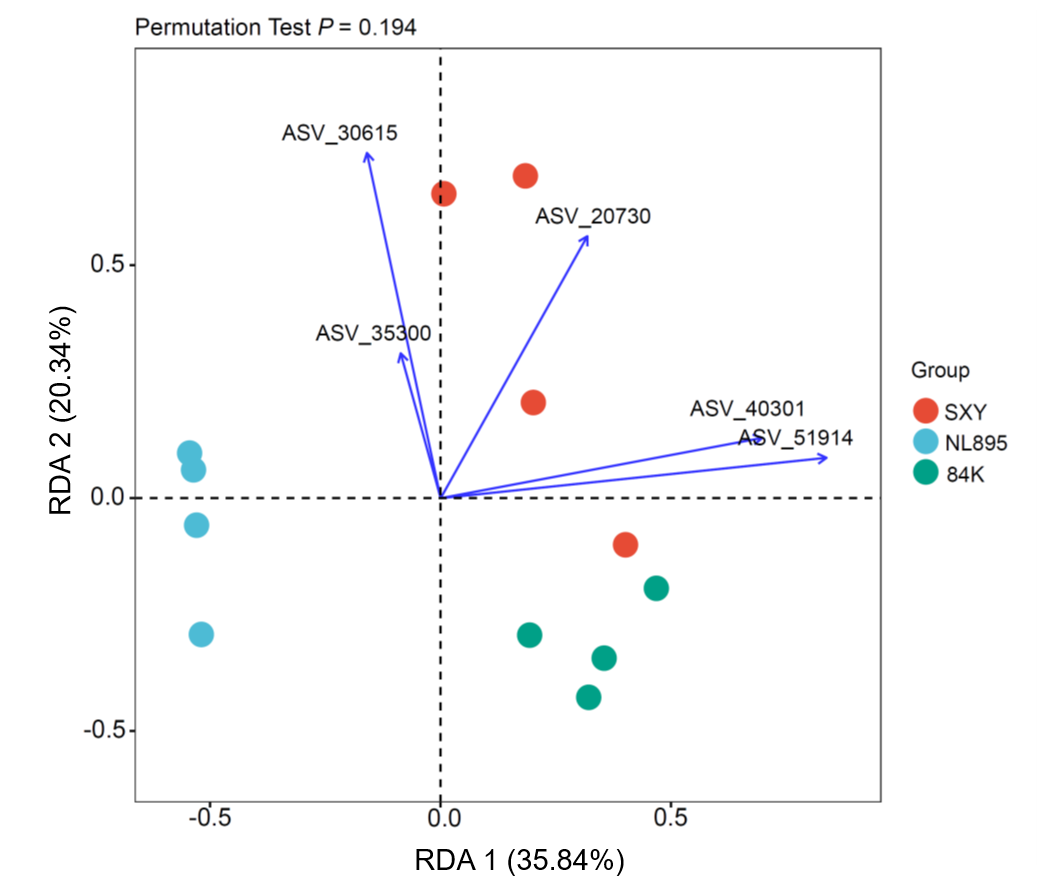
**FIG S6** RDA ordination plot illustrating the association between the root metabolites and the community structure of key microbial taxa across three poplar varieties using the abundance of microbial taxa as explanatory variables. Permutation tests were performed to assess the significance of the RDA models.
